# An Absolute Quantitative Approach to Study the Desorption Step in Plasma-Based Ambient MS Methods

**DOI:** 10.1021/acs.analchem.5c03205

**Published:** 2026-01-09

**Authors:** Odhisea Gazeli, David Moreno-González, Marcos Bouza, Charalambos Anastassiou, George E. Georghiou, Antonio Molina-Diaz, Joachim Franzke, Juan F. García-Reyes

**Affiliations:** † Analytical Chemistry Research Group, Department of Physical and Analytical Chemistry, 16747 Universidad de Jaén, Jaén 23071, Spain; ‡ Electromagnetics and Novel Applications Laboratory, Department of Electrical and Computer Engineering, 54557 University of Cyprus, Nicosia 1678, Cyprus; § FOSS Research Centre for Sustainable Energy, Department of Electrical and Computer Engineering, University of Cyprus, Nicosia 1678, Cyprus; ∥ 28371 Leibniz Institut für Analytische Wissenschaften (ISAS e.V), Dortmund 44123, Germany

## Abstract

The desorption step in ambient mass spectrometry, concerted or decoupled with ionization, triggers the transfer of a sample (analytes) from the condensed phase or surface to the gas phase. Depending on the type of method, the desorption is caused by momentum transfer, ultrasound, thermal energy, or laser pulses, among other means. In the case of plasma-based methods, thermally assisted desorption is the most commonly discussed route for analyte desorption, and although often postulated, there is no clear evidence of other mechanisms related to high-energy species created in the discharge. This study addresses the assessment of a protocol to allow absolute quantification of the desorption step during plasma-based ambient MS experiments. As a proof of principle, we measured the desorption efficiency of low-temperature plasma (LTP), which is the more widespread DBD-based ambient MS method. Model analytes such as arginine, cocaine, rhodamine G, or imazalil have been selected to quantify the desorption efficiency using 20 ng in each experiment. Two microliters of analyte solution were deposited on a glass substrate (18 mm × 18 mm) and allowed to dry. Then, they were exposed to different LTP plasma conditions (discharge gas, probe position, and desorption time). After the sample substrate was redissolved with an appropriate solvent, quantitative data were obtained using liquid chromatography/tandem mass spectrometry. Selected experiments have been completed, demonstrating the ability to quantitatively measure the amount of analyte desorbed with high precision (RSD ≤ 7%), finding subtle changes (in the absolute picomole range) when different variables such as discharge gas nature or exposure time were evaluated. Through the use of spatially resolved fluorescence microscopy measurements, we also noticed that analyte deposition is not evenly distributed on the substrate. This evidence, together with the experimental quantitative data, confirms that the conditions used to quantify the desorption are also rugged to experimental aspects such as sample deposition, since the analyte spot size interrogated (1 mm diameter) is distinctly smaller than the LTP probe diameter (4 mm i.d.) and the plasmajet area that impinges the entire sample surface. Analyte–analyte interactions and sample thickness may also be relevant in explaining the desorption in plasma-based ambient MS methods.

## Graphical abstract

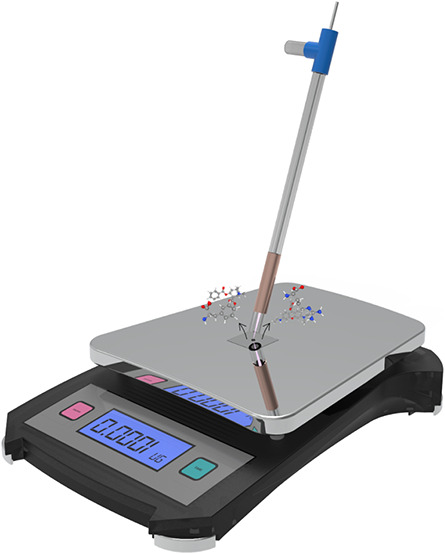

## Introduction

Ambient mass spectrometry (MS)^1,2^ refers to a set of atmospheric pressure MS methods that allow the acquisition of mass spectra on ordinary solid or liquid samples in their native environment with minimal or even no sample preparation by generating ions outside the instrument. No additional sample preparation is demanded since the sample processing takes place during the analysis through different operations—such as liquid–solid extraction in desorption electrospray ionization (DESI), liquid–liquid extraction and filtration in paper spray, thermal desorption (e.g., in direct analysis in real time (DART)), or spallation by energy sudden desorption (LAESI), each of them occurring in real-time, proximal to ionization.^3,4^ Since the deployment of ambient MS techniques, plasmas have played a significant role in the development of desorption and ionization methods.^5,6^ DART-MS^5^ and the set of dielectric barrier discharge ionization (DBDI)-based ambient MS^7^ are the most widely used and studied in the literature. DBDI setups have attracted much attention in different fields of life science due to their simplicity, flexibility, absence of solvent, and high chemical versatility. The desorption step in ambient mass spectrometry,^8^ concerted or decoupled with ionization, triggers the transfer of sample (analyte(s)) from the condensed phase or surface to the gas phase. Depending on the type of method,^7,8^ the desorption is caused by momentum transfer, ultrasounds, thermal energy, or laser pulses, among other means,^4,9,10^

While ionization mechanisms have been thoroughly studied in plasma-based ambient MS methods^6,11^ mainly via time-resolved spectroscopic plasma diagnostics,^12,13^ scarcely any literature has been devoted to understanding the desorption processes of DBD as well as other plasma-based ambient MS methods. In the case of plasma-based methods, localized surface heating is the most commonly accepted driver of desorption. Field desorption and chemical sputtering are also among the main hypothesized mechanisms, although without sound experimental support.^3,14–16^ According to Fernandez,^17^ desorption of the surface-deposited or bound analyte is generally believed to be strongly mediated by thermal desorption processes, leading to an increase in sensitivity with an increase in gas temperature at the point of desorption. This fashion holds for most low molecular weight species with *m*/*z* below 400. However, ionization in some cases can readily proceed without external heating, even for low vapor pressure analytes, indicating that additional poorly understood mechanisms are also present. Cody et al. observed that sample heating was not required for the DART ionization of two low-volatile compounds: sodium perchlorate and N,N-diisopropylaminoethyl methylphosphonothioic acid.^3^ They proposed that charged clusters might facilitate the desorption of solid analytes via a chemical sputtering mechanism.^18^ Cooks et al. reported the effective direct ionization of low vapor pressure nitroaromatic explosives either directly by the LTP plasma or when the discharge gas was doped with charged solvent vapors.^19^ Additional experiments with self-assembled monolayers also suggested the route of chemical sputtering.^20^

Farnsworth et al.^21^ studied the effect of adding H_2_ as a dopant in discharge gases (argon and helium) to enhance both desorption and ionization steps in a DBD-based ambient ionization probe. They found an increase in signal and a shift in the signal time profile that could not be adequately described with a simple thermal model involving added H_2_. A follow-up study^22^ confirmed the lack of correlation between the measured surface temperature and the signal intensity and time profile, suggesting the contribution of a nonthermal component to the desorption process when adding Ar or hydrogen as dopants (1–3%) to the discharge gas (helium). Possible speculated mechanisms were chemical sputtering and field desorption, although no further studies were conducted. None of these studies separates the contribution of the discharge gas composition effects on the desorption and ionization steps. As for the experimental methods used for desorption mechanistic studies with plasma-based techniques and DESI, they were mainly based on (1) total ion current measurements of the entire process so that the ionization step was not excluded, unlike in other mechanistic studies where each contribution was decoupled;^23^ and (2) the use of dyes (coumarin-coated substrate) and visual inspection (or with a microscope) after analysis.^24,25^ Notably, Bereman and Muddiman used the fluorescence intensity before and after DESI analysis of rhodamine 6G to determine the amount of material removed from the surface per mass spectrum, leading to low attomole amounts removed per linear ion trap spectrum.^26^

Most of these studies share the limitation of assessing the desorption and ionization effects using MS data, which account for only the fraction of ionized species and cannot provide a precise measurement of the desorption step. The direct data from the MS instrument are used with a somewhat limited linear response and precision of the detector, which makes it difficult to extract meaningful conclusions from the data with regard to desorption solely. In this work, we propose a method to quantitatively study the desorption step in plasma-based ambient MS methods, decoupling the ionization efficiency and ion detection contribution/impact. It is based on the realization of a precise and absolute mass balance, completed by highly sensitive batch liquid chromatography (LC)-MS/MS measurements of the analyte (deposited on the substrate) before and after the surface is subjected to the plasmajet stream. The novel aspect of the proposed approach is the ability to generate quantitative data on desorption rates to gain insight into the desorption mechanisms of plasma-based ambient MS methods and the main parameters that may play a relevant role. The use of an absolute approach, measuring the entire spot after plasma treatment of the surface, enables the quantitation of the desorbed analyte in a reproducible fashion, even if the sample deposition is not homogeneous. Examples are shown, illustrating the type of information that can be gathered to understand the effect of the different parameters and set the basis for improved methods or tailored applications where the desorption step can be considered critical.

## Experimental Section

### Chemicals, Reagents, and Apparatus

LC-MS grade water and methanol were purchased from Merck (Darmstadt, Germany). Analytical standards (imazalil, cocaine, phenylalanine, arginine, tylosin, and rhodamine 6G) were obtained from Sigma-Aldrich (Madrid, Spain) and Cerilliant (Round Rock, TX). Solutions were prepared at 500 ng μL^–1^ in methanol and diluted with methanol/water (50:50, v/v) to the desired concentrations. Helium 5.0, Argon 5.0 (99.999%) (ALPHAGAZ grade), and synthetic air (ALPHAGAZ grade) were supplied by Air Liquide (Madrid, Spain). Microscope glass slides (18 × 18 mm, plain glass), supplied by Epredia (Kalamazoo, MI, USA), were used as substrates throughout the experiments with no further treatment. Before use, the glass substrates were rinsed with methanol and allowed to dry at room temperature. A 4-L ultrasound bath from JP Selecta was also used (Thermo Fisher, Madrid, Spain).

### Low-Temperature Plasma (LTP) Desorption/Ionization Probe

An LTP probe was used to evaluate the proposed quantitative approach. The LTP probe setup is described elsewhere^27^ (Figure 1). An AC voltage of 6.2 kV at a frequency of 2.7 kHz is typically applied to the outer electrode, with the center electrode grounded to generate the nonequilibrium (low-temperature) discharge. A sinusoidal waveform generator was coupled to a power amplifier and an automobile engine ignition coil to provide an AC voltage with an amplitude as high as 11.2 kV. Helium 5.0, Argon 5.0, and synthetic air were used as discharge gases at a flow rate of 0.40 L min^–1^. The LTP probe was placed with its end 10 mm away from the surface at a 45° angle to the sample surface. The LTP probe was mounted on an *x*, *y*, and *z* manual linear stage to control the probe position and distances to the glass substrate. The microscope glass slides were positioned on the top of an 18 mm × 18 mm quad-ruled polymer sheet with marks indicating where the sample should be deposited so that the desorption experiments were performed in a reproducible fashion.

**Figure 1:**
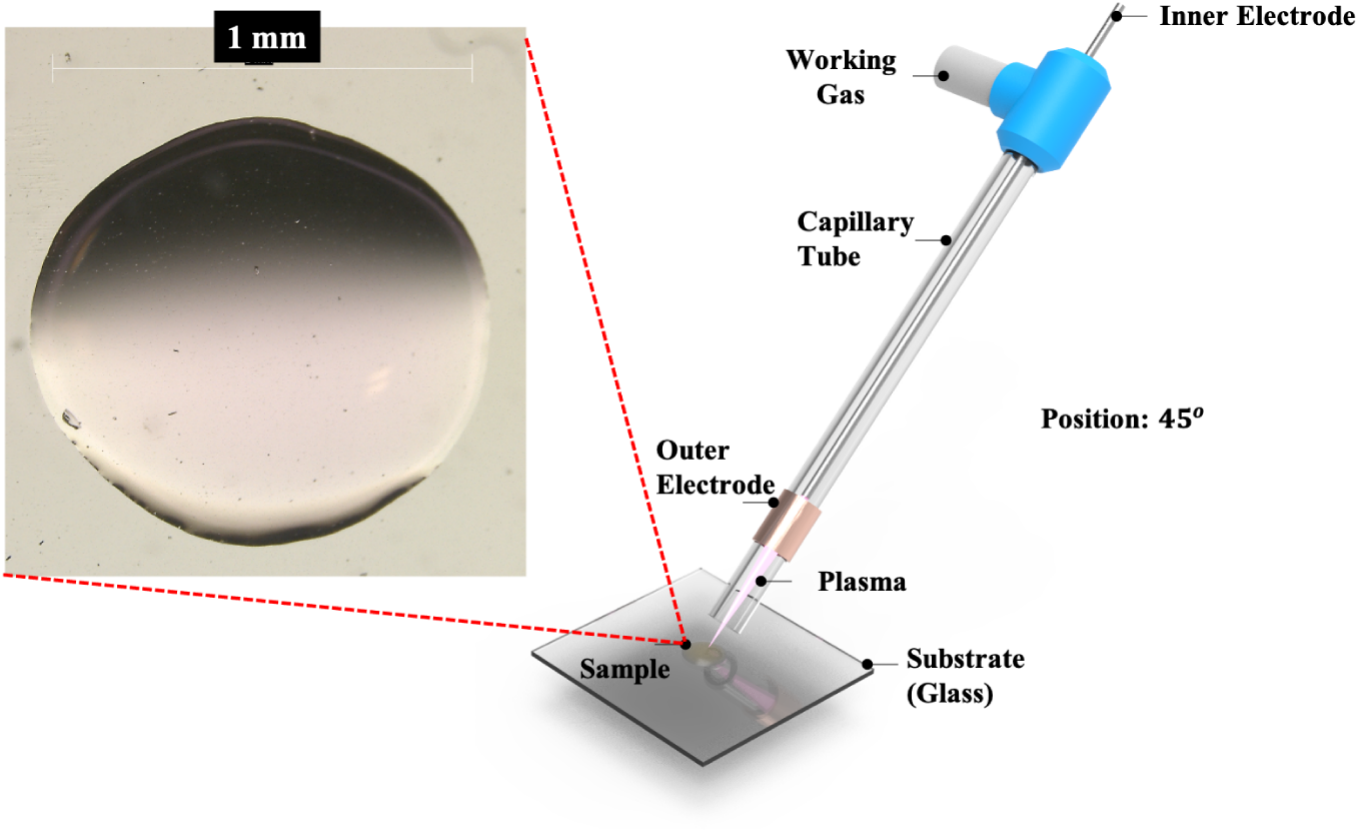
Schematic view of the desorption experiment using an LTP probe.

### Ultra-high Pressure Liquid Chromatography Tandem Mass Spectrometry (LC-MS/MS )

LC-MS/MS acquisitions were performed with a Dionex Ultimate 3000 UHPLC system (Thermo Fisher Scientific, Waltham, MA, USA) coupled to a TSQ Quantiva triple quadrupole mass spectrometer (Thermo Fisher Scientific, San José, CA, USA) using electrospray ionization (ESI) in positive ion mode. Data acquisition and processing for analyte confirmation and quantitative analysis were carried out using the Xcalibur 3.0 and TraceFinder 3.3 software packages (Thermo Scientific). UHPLC separation was performed using a reverse-phase C18 column (Zorbax Rapid Resolution High Definition (RRHD) Eclipse Plus C18 (2.1 × 100 mm, 1.8-μm particle size; Agilent Technologies, Santa Clara, CA, USA)), whose temperature was kept constant at 25 °C throughout the analysis. The mobile phases were water (solvent A) and MeCN (solvent B), both with 0.1% formic acid. The gradient elution program was as follows: 0 min, 5% B; 1.0 min, 5% B; 7.0 min, 70% B; 10.0 min, 95% B; 12.0 min, 95% B; 12.5 min, 5% B; 16 min, 5% B. The flow rate was 0.4 mL min^–1^, and the injection volume was 5 μL. All studied analytes were detected in the positive ionization mode using multiple reaction monitoring (MRM) acquisition mode or selected ion monitoring MS mode. The main ion source parameters were as follows: spray voltage of 3500 V; sheath gas of 45 arbitrary units (a.u.); aux gas of 10 a.u.; sweep gas: 0 a.u. Other relevant parameters were as follows: ion transfer tube temperature, 350 °C; vaporizer temperature, 350 °C; collision gas (CID), 1.5 mTorr.

### Stepwise Assay for the Quantitation of the Desorption Step in Ambient MS Methods

Model analytes (imazalil, cocaine, arginine, phenylalanine, rhodamine 6G, and tylosin) have been used to study the impact of different parameters on desorption efficiency using 20 ng of analyte in each assay. The assay consisted of four steps (Figure 2). LC-MS/MS measurements were carried out to account for any losses of analyte during spotting and drying, and also to decouple the contribution from neutral desorption processes caused by the gas stream. The steps are described as follows:

(1) Quality control/reference signal with no analyte loss. A standard with a variable amount of analyte (e.g., 20 ng) is prepared by pipetting 2 μL of the working solution and diluting it up to 5 mL with H_2_O/MeOH (50:50, v/v), and tested by LC-MS/MS with the detailed procedure.
(2) Sample deposition, drying, and dissolution. The same amount of analyte (2 μL) is spotted on a microscope glass slide and left to dry for 1 h. Then, the dried slide is placed in a beaker, and a 5 mL aliquot of solvent (H_2_O/MeOH (50:50, v/v)) is added. The content is homogenized using an ultrasound bath for 5 min. A 1 mL aliquot is then transferred to a 2 mL glass vial and subjected to LC-MS/MS analysis.
(3) Control experiment with flowing gas, but with no plasma ignited. The same amount of analyte (2 μL) is spotted on the glass substrate and left to dry for 1 h. Then, the dried substrate is interrogated by the LTP probe with the discharge gas flow (0.4 L min^–1^ of helium, argon, or air) but without igniting the plasma, just accounting for any possible neutral desorption effect during the study period . Then, the dried slide is placed in a beaker, and a 5 mL aliquot of solvent is added, with the solution homogenized using an ultrasound bath for 5 min. A 1 mL aliquot is then transferred to a 2 mL glass vial and subjected to LC-MS/MS analysis.
(4) Desorption experiment with the substrate exposed to the *plasmajet*. The exact amount of analyte (2 μL) is spotted and left to dry for 1 h. Then, the dried substrate is interrogated by the ignited LTP probe for the analyte desorption study. Then, the dried slide is placed in a beaker, and a 5 mL aliquot of solvent is added, with the solution homogenized using an ultrasound bath for 5 min. A 1 mL aliquot is then transferred to a 2 mL glass vial and subjected to LC-MS/MS analysis.

**Figure 2:**
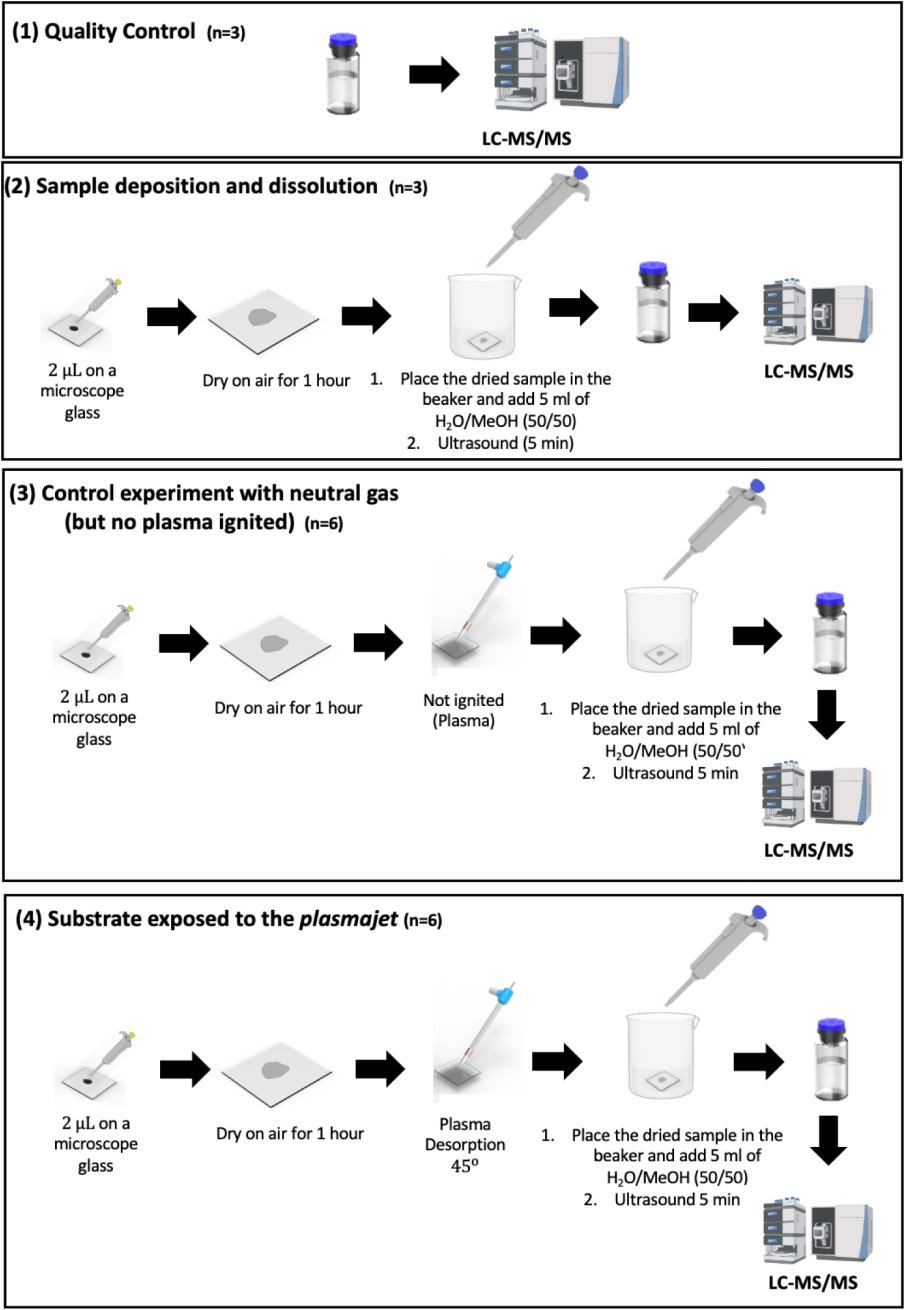
Scheme of the proposed approach to study the desorption step in plasma-based ambient MS methods. For details, see the Experimental Section.

### Fluorescence Microscopy Measurements

Sample drop micrographs were acquired on an Olympus BX51 fluorescence microscope (model BX51TF) with an Olympus USPT trinocular head (Japan) and an Olympus Plan N 10×/0.25 NA objective. Fluorescence excitation was provided by a 100 W mercury lamp (Olympus ULH100HG) powered by an Olympus URFLT unit. Rhodamine (20 ng, 2 μL of a 10 ng μL^–1^ methanol solution) was used as the fluorophore, and emission was recorded in the green channel. Images were captured via the trinocular port using a digital camera and DPController software, version 1.1.1.65 (Olympus Optical Co., Ltd.).

## Results and Discussion

Different discharge gases (helium, argon, and synthetic air) were studied together with the desorption time and other electrical and geometrical parameters. Selected experiments have been completed using pesticides, amino acids, and other compounds of interest, demonstrating the ability to quantitatively measure the amount of analyte desorbed, and finding subtle changes when different variables, such as probe angle, discharge gas nature, or exposure time, are modified. The data from some of the experiments are discussed.

### Study of Desorption Variables: Exposure Time to *Plasmajet*

An interesting experiment to show the ability of the proposed approach to provide insights into the desorption event was the study of exposure time, carried out with imazalil (20 ng) using Helium 5.0 as the discharge gas and 1, 3, 6, and 10 min as exposure times (Figure 3). The results from the stepwise (step 4) experiment are shown in Figure 3a, corresponding to the lowest trace to the amount measured in the substrate after desorption (step 4). Figure 3b shows the replicates (*n* = 6) of the measured analytes after plasma desorption for 3 min with RSD values of peak area below 7% (Table 1). Figure 3c illustrates that increased exposure times led to a higher amount of the analyte desorbed from the surface. However, there is no linear tendency, and the amount of desorbed material nearly reaches a plateau after 6 min. Note that the surface area impinged by the plasma (provided the large diameter of the LTP probe (4 mm i.d.) and the gas flow rates) is distinctly higher than the footprint of the deposited (2 μL) sample (1 mm diameter circle) (Figure 1). So, all analytes were “macroscopically” exposed to the plasma at all times. Interestingly, this decay effect could be explained by hotspots or a “sweet spot effect” due to the uneven surface charge accumulation across the substrate area impinged by the plasma. The analyte desorbed would be from localized areas where all of the analytes sre released, while other areas may remain intact, provided the analyte fraction was left after the desorption experiment. This observation would be consistent with the fact that the LTP probe, operated at relatively high voltages (above 5 kV), features a nonhomogeneous, filamentary-type discharge with localized streamers.^28^ A recent study using COMSOL simulations found that charges accumulate in the sample substrate and may result in electric fields strong enough to disrupt analyte–substrate interactions, thus enhancing desorption.^29^

**Figure 3:**
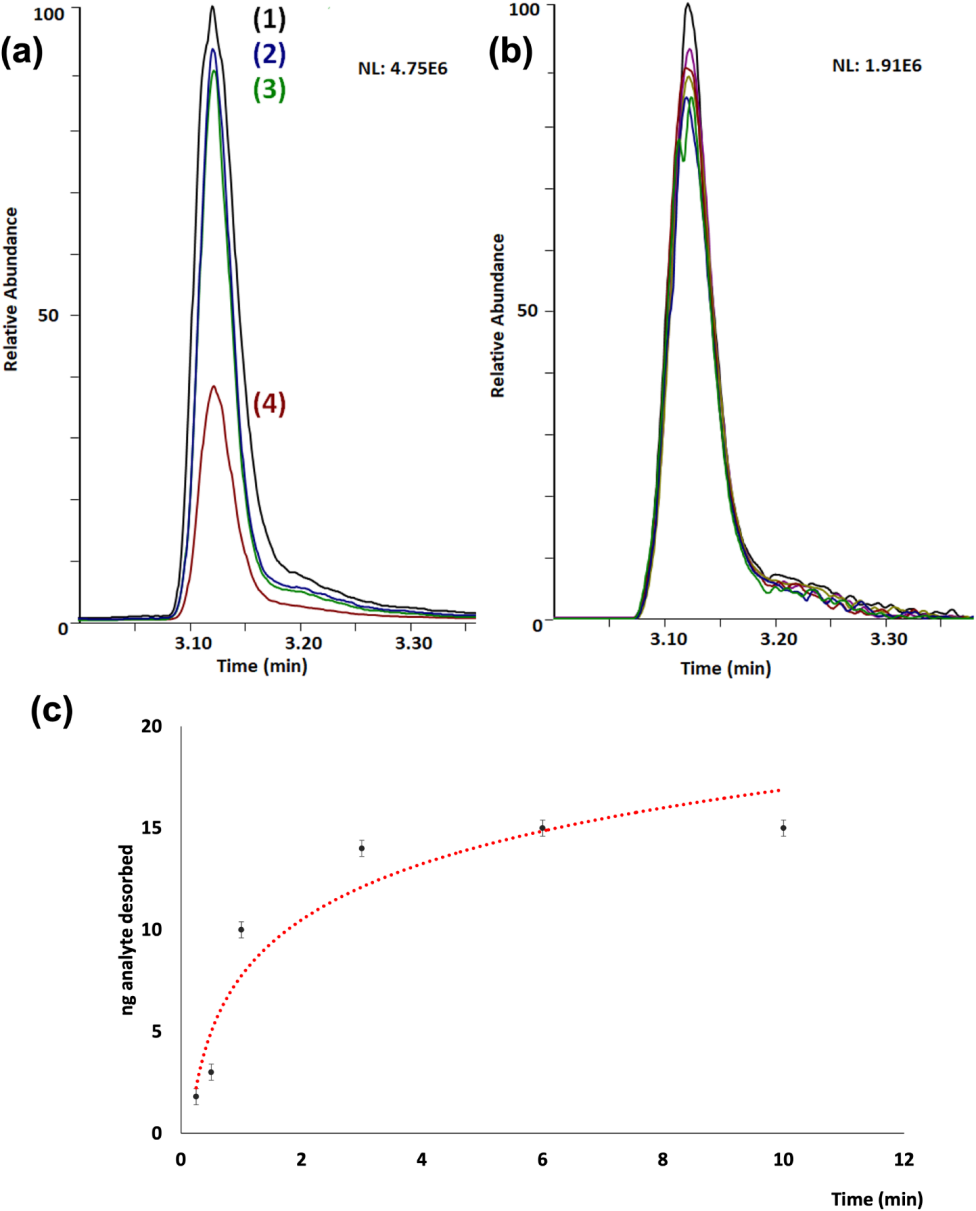
(a) Example of a desorption study. Extracted ion chromatograms of the different steps of the experiment: (1) quality control; (2) sample deposition and dissolution; (3) control experiment with gas (plasma not ignited); and (4) sample exposed to the ignited plasma. (b) Replicates (*n* = 6) from the desorption experiment (step 4) (RSD 6.1%). (c) Study of different exposure times for the desorption of imazalil (20 ng deposited). Increased exposure to the DBD plasma jet leads to higher absolute amounts of desorbed analyte, expressed in nanograms of the desorbed analyte (imazalil).

**Table 1:** Effect of Exposure Time to Plasmajet for 20 ng of Deposited Imazalil^a^

	Quality control (QI)	Dry and redissolve (*n* = 6)		Control experiment without igniting the plasma (*n* = 6)		Desorption experiment (*n* = 6)	
Time (min)	Normalized signal (RSD %)	Normalized signal (RSD %)	Amount lost (ng)	Normalized signal (RSD %)	Amount desorbed (ng)	Normalized signal (RSD %)	Amount desorbed (ng)
1 min	100% (2.5%)	93% (3.6%)	1.4	93% (3.8%)	1.4	49% (6.0%)	10.3
3 min	100% (2.4%)	93% (3.4%)	1.4	91% (3.3%)	1.8	32% (6.1%)	13.6
6 min	100% (2.4%)	93% (3.4%)	1.2	90% (2.4%)	2.0	27% (6.0%)	14.7
10 min	100% (2.4%)	93% (3.7%)	1.4	90% (2.9%)	2.0	25% (5.3%)	15.0

a Discharge gas: helium; discharge gas flow rate: 400 mL min^–1^; angle: 45°.

However, experimental data harnessing the native fluorescence of rhodamine G were collected to provide spatially resolved information on the desorption efficiency through fluorescence microscopy. We first confirmed that the solution evaporation is not uniform with selected areas where the analyte is concentrated during evaporation (Figure 4). These areas were not successfully desorbed compared to regions with homogeneously distributed rhodamine G. So, the intermolecular analyte–analyte interactions and the thickness of the sample are also parameters that may play an important role in the desorption process. The fluorescence microscopy measurements provide additional evidence to support the discussion of the desorption saturation reported. This saturation cannot be attributed to the nature of the plasma, but rather to the uneven distribution of the evaporated sample. This evidence, together with the experimental quantitative data, also confirms that the conditions used to quantify the desorption are also rugged against experimental aspects such as sample deposition, since the area of the sample drop deposited (1 mm diameter) is distinctly smaller than the LTP probe diameter (4 mm i.d.) and thus the actual plasmajet area impinges on the entire sample surface.

**Figure 4:**
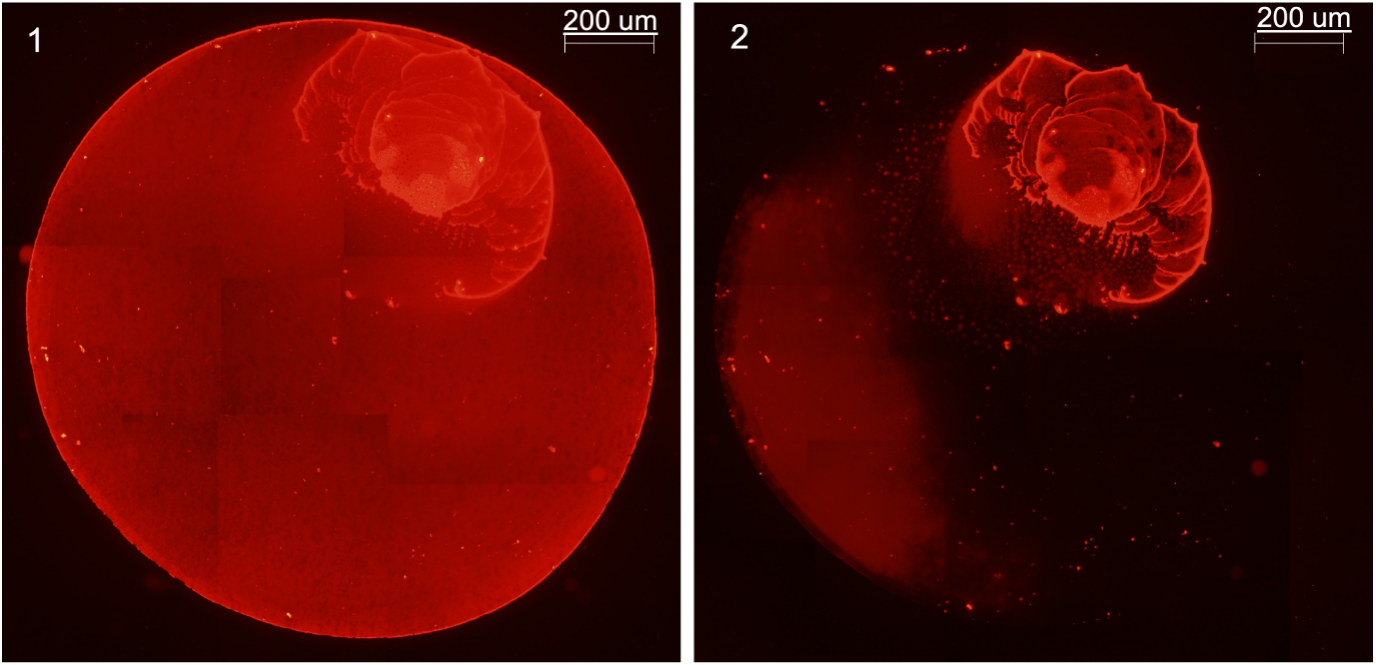
Fluorescence microscopy images from a 2 μL droplet of 10 ng μL^–1^ rhodamine G solution (20 ng) dried for 1 h on a microscope glass slide (1) and (2) subjected to desorption for 5 min with a helium LTP plasma (placed on the right of the glass slide, at an angle of 45° with respect to the surface (see Figure 1)). During evaporation, the analyte is not evenly distributed, eventually concentrating in a reduced portion of the total substrate area where the solution is initially placed (1 mm diameter circle). After the 5 min desorption with LTP, most of the substrate areas treated, particularly the areas where rhodamine G was homogeneously distributed, no longer fluoresces, indicating the complete desorption of the analyte. In contrast, the desorption is not comprehensive in the sections where rhodamine G is concentrated during evaporation. This evidence suggests that analyte intermolecular interactions and the thickness of the sample layer are also parameters that may play an important role in the desorption process.

### Study of Discharge Gases

The composition of the gas used as the discharge gas is relevant for both desorption and ionization steps. Some studies have used the controlled addition of impurities as dopants to increase ionization^30,31^ and desorption,^21,22,25^ in different ambient MS methods with variable results and outcomes. In this study, we aimed to isolate the effect of the discharge gas composition on the desorption efficiency using an LTP probe with three common gases (helium, argon, and synthetic air). Data obtained from the study of different discharge gases are summarized in Table 2. Each experiment was conducted at the minimum amplitude voltage that ignited the plasma with each discharge gas composition (6 kV for Ar and He, and 11.2 kV for air). We tested two different compounds, imazalil and arginine, with opposite trends and slight differences in terms of desorption under the conditions tested. In the case of the data acquired from imazalil, the amount desorbed with Ar plasma was higher than those with air and helium. This trend was discussed previously by other authors who attributed a higher momentum (of the heavier gas) to yield higher desorption.^17^ Farnsworth and Venter have described similar results in terms of desorption and ionization efficiency for DBD and DESI ambient methods,^21,22,25^ In contrast, in the case of arginine, 15% of the analyte was desorbed with helium, 11% with Ar, and 8% with air. The strong surface interaction of arginine with the silanol group from the glass substrate surface through hydrogen bonds might be contributing to this pattern,^32^ with low desorption efficiencies regardless of the discharge gas used. This illustrates the central effect of the analyte structure and properties (vapor pressure, dielectric constant, etc.) and its possible interaction with the substrate.

**Table 2:** Effect of the Type of Discharge Gas on the Desorption of 20 ng of Deposited Imazalil^a^

	Quality control (QI)	Dry and redissolve (*n* = 6)		Control experiment without igniting the plasma (*n* = 6)		Desorption experiment dried spot exposed to the plasma (*n* = 6)	
	Normalized signal (RSD, %)	Normalized signal (RSD, %)	Amount lost (ng)	Normalized signal (RSD, %)	Amount desorbed (ng)	Normalized signal (RSD, %)	Amount desorbed (ng)
Helium	100% (2.4%)	93% (3.4%)	1.4	91% (3.3%)	1.8	32% (6.1%)	13.6
Argon	100% (2.9%)	94% (3.0%)	1.2	89% (3.5%)	2.2	23% (5.9%)	15.4
Air	100% (2.9%)	95% (3.1%)	1.0	93% (3.3%)	1.4	33% (7.0%)	13.4

a Exposure time: 3 min; discharge gas flow rate: 400 mL min^–1^; angle: 45°.

### Selected Examples of Different Desorption Patterns Depending on the Nature of the Compound

A set of compounds with different physicochemical properties and a wide range of molecular weights was selected. Detailed data from selected experiments and the associated precision data are listed in Table 3. Diagram bars from the comparison of the different steps of the desorption study for the selected model compounds are shown in Figure 5. First, two examples of compounds with effective desorption are imazalil (Figure 5a) and cocaine (Figure 5b). Both species exhibited relatively high desorption rates, with data consistent with the ability to measure both compounds by LTP-MS without additional substrate heating.^33,34^ Arginine and phenylalanine (Mw = 165.2; C_9_H_11_NO_2_) (Figure 5c and d) were selected as representative compounds with low desorption rates in plasma-based ambient methods.^35^ Amino acids, despite their relatively low molecular weight, exhibit low vapor pressure and tend to interact with glass through hydrogen bonding (silanol-amine moieties) or nonselective adsorption events.^36^ In addition, the dye rhodamine 6G (Mw = 479.01; C_28_H_31_N_2_O_3_) and the macrolide antibiotic tylosin (Mw = 917; C_46_H_77_NO_17_) were expected to have lower desorption efficiency due to their relatively high molecular weight. The desorption exhibited by rhodamine 6G (Figure 5e) is consistent with the relatively low vapor pressure and high molecular weight. The contribution from neutral desorption was found to be higher (two-thirds of the desorbed analyte) than that from the actual *plasmajet* exposure (one-third of the total amount of the analyte desorbed). Finally, tylosin (Figure 5f) also exhibited a similar desorption pattern with a minimal desorption efficiency. LTP-MS cannot detect any of these compounds without external heating assistance. Furthermore, considering a confidence interval of 1.96SD (95% probability), there would not be meaningful differences in the desorption with and without the discharge.

**Table 3:** Study of the Desorption Efficiency of Different Compounds Using an LTP Probe

	(1) Quality control (*n* = 3)^a^	(2) Sample deposition and dissolution (*n* = 6)		(3) Control experiment with neutral gas (no plasma ignited) (*n* = 6)		(4) Substrate exposed to the plasma (*n* = 6)^b^	
	Normalized signal (RSD %)	Normalized signal (RSD, %)	Amount lost (ng)	Normalized signal (RSD %)	Amount desorbed (ng)	Normalized signal (RSD, %)	Amount desorbed (ng)
Imazalil	100% (2.4%)	93% (3.4%)	1.4	91% (3.3%)	1.8	32% (6.1%)	13.6
Cocaine	100% (2.2%)	96% (3.9%)	0.8	89% (3.8%)	2.2	59% (6.0%)	8.2
Arginine	100% (2.4%)	93% (3.2%)	1.4	78% (5.4%)	4.4	72% (4.7%)	5.6
Phenylalanine	100% (3.4%)	96% (4.6%)	0.8	91% (6.5%)	1.8	75% (7.8%)	5.0
Rhodamine G	100% (2.6%)	96% (4.6%)	0.8	85% (5.8%)	3.0	81% (7.8%)	3.8
Tylosin	100% (2.1%)	97% (2.9%)	0.6	91% (3.1%)	1.8	88% (4.3%)	2.4

a 20 ng of analyte was deposited in all experiments.

b Desorption time: 3 min; discharge gas: He; discharge gas flow rate: 400 mL min^–1^; angle: 45°.

**Figure 5:**
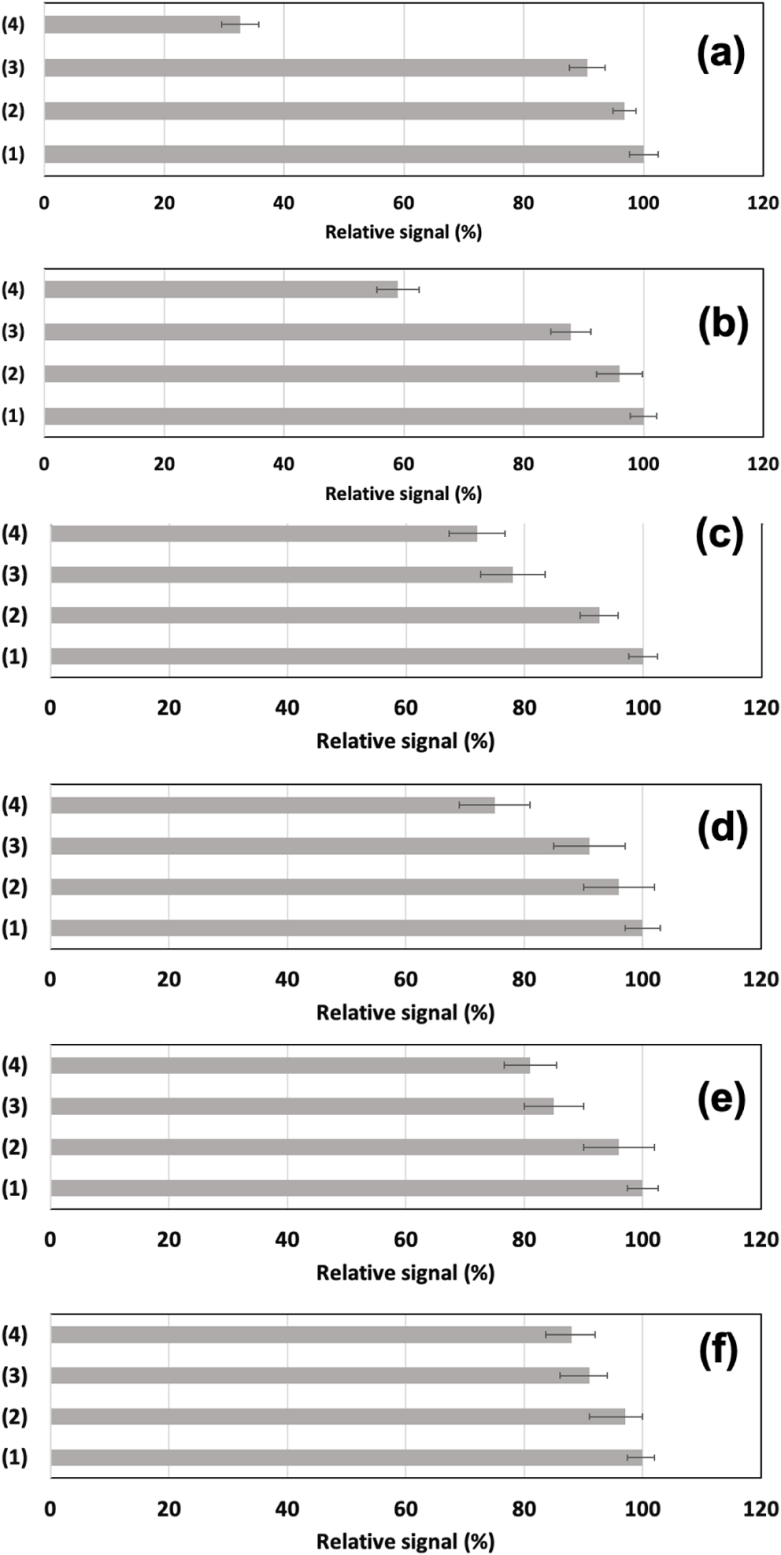
Desorption experiments with different compound classes: (a) imazalil; (b) cocaine; (c) arginine; (d) phenylalanine; (e) rhodamine; and (f) tylosin. All experiments were carried out with 20 ng of analyte deposited on the substrate. The experiment number refers to (1) quality control experiment (reference signal); (2) sample deposition and dissolution; (3) control experiment with neutral gas (but no plasma ignited); and (4) substrate exposed to the plasma for 3 min. For details, see the text.

## Concluding Remarks

The proposed approach generates useful experimental data for desorption rates. The gathered data provide essential information not only to understand the mechanism by which plasma-based ambient ionization sources operate but also for the optimization of their performance for mass spectrometric analysis. The final aim of this approach is to study the plasma conditions using DBDs that lead to improved desorption and also to decipher which are the actual species or phenomena primarily involved in the desorption step of DBD plasma-based ambient ionization methods. Not only plasma-related aspects such as discharge gas, amplitude voltage, or the actual position of the probe but also substrate-related and analyte-related aspects are considered. Among them, and besides the nature of the analyte itself, analyte–analyte interactions and sample thickness may also be relevant in explaining the desorption in plasma-based ambient MS methods. These roles might be deciphered by combining plasma diagnostic tools (e.g., time-resolved and temporally resolved spectroscopic emission measurements) with the actual quantitative data from the desorption efficiency and the use of spatially resolved tools such as fluorescence microscopy images of key native fluorescent analytes.

## References

[1] Takats Z., Wiseman J. M., Gologan B., Cooks R. G. (2004). Mass spectrometry
sampling under ambient conditions with Desorption Electrospray Ionization. Science.

[2] Cooks R. G., Ouyang Z., Takats Z., Wiseman J. M. (2006). Ambient Mass Spectrometry. Science.

[3] Venter A. R., Douglass K. A., Shelley J. T., Hasman G., Honarvar E. (2014). Mechanisms of Real-Time, Proximal Sample Processing
during Ambient ionization Mass Spectrometry. Anal. Chem..

[4] Javanshad E., Venter A. R. (2017). Ambient ionization
mass spectrometry: real-time, proximal
sample processing and ionization. Anal. Methods.

[5] Cody R. B., Laramee J. A., Durst H. D. (2005). Versatile
new ion source for the
analysis of materials in open air under ambient conditions. Anal. Chem..

[6] Chen J., Tang F., Guo C., Zhang S., Zhang X. (2017). Plasma-based
ambient mass spectrometry: a step forward to practical applications. Anal. Methods.

[7] Dryahina, K. ; Polasek, M. ; Jasik, J. ; Sovoka, K. ; Spanel, P. Ion chemistry in Dielectric Barrier Discharge Ionization: Recent Gas Advances in Direct Phase Analyses. Mass Spectrom. Rev., 2024, 10.1002/mas.21914. PMC12866379 39506464

[8] Usmanov D. T., Ninomiya S., Chen L. C., Saha D., Mandal M. K., Sakai Y., Takaishi R., Habib A., Hiraoka K., Yoshimura K., Takeda S., Wada H., Nonami H. (2017). Desorption
in Mass Spectrometry. Mass Spectrom..

[9] Chen X., Newsome G. A., Buchanan M., Glasper J., Hua L., Laftif M., Gandhi V., Li X., Larriba-Andaluz C. (2023). Flow optimized
model for gas jet desorption sampling mass spectrometry. J. Phys. Chem. A.

[10] Guo G., Tang F., Chen J., Wang X., Zhang S., Zhang X. (2015). Development
of dielectric barrier discharge ionization. A review. Anal. Bioanal. Chem..

[11] Pape A., Schmitz O. J. (2024). Dielectric barrier discharge in mass
spectrometry.
An overview over plasma investigations and ion source applications. Trends Anal. Chem..

[12] Chan G. C.-Y., Shelley J. T., Wiley J. S., Engelhard C., Jackson A. U., Cooks R. G., Hieftje G. M. (2011). Elucidation of reaction
mechanisms responsible for afterglow and reagent-ion formation in
the low-temperature plasma probe ambient ionization source. Anal. Chem..

[13] Klute F. D., Brandt S., Franzke J. (2021). Spatiotemporal characterization of
different dielectric barrier discharges designed for soft ionization. Spectrochim. Acta Part B.

[14] Albert A., Shelley J. T., Engelhard C. (2014). Plasma-based
ambient desorption/ionization
mass spectrometry: state-of-the-art in qualitative and quantitative
analysis. Anal. Bioanal. Chem..

[15] Martínez-Jarquín S., Winkler R. (2017). Low-temperature plasma (LTP). jets for mass spectrometry
(MS): ion processes, instrumental set-ups and application examples. Trends Anal. Chem..

[16] Chan G. C.-Y., Shelley J. T., Jackson A. U., Wiley J. S., Engelhard C., Cooks R. G., Hieftje G. M. (2011). Spectroscopic plasma
diagnosis on
a low-temperature plasma probe for ambient mass spectrometry. J. Anal At. Spectrom..

[17] Monge M. E., Harris G. A., Dwivedi P., Fernandez F. M. (2013). Mass Spectrometry:
Recent Advances in Direct Open Air Surface Sampling/Ionization. Chem. Rev..

[18] Cooks R. G., Ast T., Pradeep T., Wysocki V. (1994). Reactions of Ions with organic surfaces. Acc. Chem. Res..

[19] García-Reyes J. F., Harper J. D., Salazar G. A., Charipar N. A., Ouyang Z., Cooks R. G. (2011). Detection of explosives and related compounds by low-temperature
plasma ambient ionization mass spectrometry. Anal. Chem..

[20] Wiley, J. S. Instrumentation, Applications and fundamentals of plasma ionization of organic molecules from surfaces; Purdue University, Purdue e-Pubs, Ph.D. Thesis, 2013.

[21] Wright J. P., Heywood M. S., Thurston G. K., Farnsworth P. B. (2013). The effects
of added hydrogen on a helium atmospheric-pressure plasma jet ambient
desorption/ionization source. J. Am. Soc. Mass
Spectrom..

[22] Ellis W. C., Lewis C. R., Openshaw A. P., Farnsworth P. B. (2016). The effects
of added hydrogen on noble gas discharges used as ambient desorption
ionization sources for mass spectrometry. J.
Am. Soc. Mass Spectrom..

[23] Douglass K. A., Jain S., Brandt W. R., Venter A. R. (2012). Deconstructing
Desorption
Electrospray Ionization: Independent Optimization of Desorption and
Ionization by Spray Desorption Collection. J.
Am. Soc. Mass Spectrom..

[24] Javanshad R., Panth R., Maser T. L., Venter A. R. (2022). Helium
assisted
desorption and spray ionization. Int. J. Mass
Spectrom..

[25] Binney B., Joseph G., Venter A. (2024). Hydrogen is
the Superior Nebulization
Gas for Desorption and Electrospray Ionization. Anal. Chem..

[26] Bereman M.
S., Muddiman D. C. (2007). Detection
of attomole amounts of analyte by desorption
electrospray ionization mass spectrometry (DESI-MS). determined using
fluorescence spectroscopy. J. Am. Soc. Mass
Spectrom..

[27] Harper J. D., Charipar N. A., Mulligan C., Ouyang Z., Zhang X., Cooks R. G. (2008). Low-temperature plasma probe for ambient desorption
ionization. Anal. Chem..

[28] Meyer C., Müller S., Gilbert-López B., Franzke J. (2013). Impact of
homogeneous and filamentary discharge modes on the efficiency of dielectric
barrier discharge ionization mass spectrometry. Anal. Bioanal. Chem..

[29] Gazeli, O. ; Lazarou, C. ; Bouza, M. ; Moreno-González, D. ; Anastassiou, C. ; Franzke, J. ; Garcia-Reyes, J. F. ; Georghiou, G. Insight Into The Desorption Mechanisms Of Plasma-Based Ambient Ionization Methods Using Computational Simulations. (submitted). 10.1021/jasms.5c00171 PMC12492398 40937483

[30] Schütz A., Lara-Ortega F. J., Klute F. D., Brandt S., Schilling M., Michels A., Veza D., Horvatic V., García-Reyes J. F., Franzke J. (2018). Soft Argon-Propane Dielectric Barrier Discharge. Anal. Chem..

[31] Vogel P., Marggraf U., Brandt S., García-Reyes J. F., Franzke J. (2019). Analyte-Tailored Controlled Atmosphere improves dielectric
barrier discharge ionization mass spectrometry performance. Anal. Chem..

[32] Rimola A., Sodupe M., Ugliego P. (2009). Affinity Scale for
the interaction
of Amino Acids with Silica Surfaces. J. Phys.
Chem. C.

[33] Jackson A.
U., Garcia-Reyes J. F., Harper J. D., Wiley J. S., Molina-Díaz A., Ouyang Z., Cooks R. G. (2010). Analysis of drugs of abuse in biofluids
by low-temperature plasma (LTP). ionization mass spectrometry. Analyst.

[34] Wiley J. S., García-Reyes J. F., Harper J. D., Charipar N. A., Ouyang N., Cooks R. G. (2010). Screening of agrochemicals in foodstuffs
using low-temperature plasma (LTP). ambient ionization mass spectrometry. Analyst.

[35] Albert A., Engelhard C. (2012). Characteristics of Low-Temperature Plasma Ionization
for Ambient Mass Spectrometry Compared to Electrospray Ionization
and Atmospheric Pressure Chemical Ionization. Anal. Chem..

[36] Xu X., Lott J., Kelly K. A., Shi Z. (2022). Adsorption of amine
compounds on the glass surface and their impact on the development
of analytical method and pharmaceutical process. Org. Process Res. Dev..

